# Selenium Uptake by Lettuce (*Lactuca sativa* L.) and Berseem (*Trifolium alexandrinum L.*) as Affected by the Application of Sodium Selenate, Soil Acidity and Organic Matter Content

**DOI:** 10.3390/plants9050605

**Published:** 2020-05-09

**Authors:** Myrto Tsioubri, Dionisios Gasparatos, Maria Economou-Eliopoulos

**Affiliations:** 1 Department of Natural Resources Management and Engineering, Agricultural University of Athens, Iera Odos 75, 11855 Athens; Greece; myrtotsioubri@aua.gr; 2 Faculty of Geology and Geoenvironment, National and Kapodistrian University of Athens, Zografou, 15784 Athens, Greece; econom@geol.uoa.gr

**Keywords:** selenium, soil properties, pH, organic matter, sodium selenate

## Abstract

Selenium deficiency in humans and animals can be reduced through dietary supplementation. Therefore, Se biofortification strategy is important in food plants and pastures. In this study, the effects of selenium (Se) addition (4 mg Se/kg) as sodium selenate (Na_2_SeO_4_) on lettuce (*Lactuca Sativa* L.) and berseem cultivation (*Trifolium alexandrinum* L.) were investigated. The experiment was conducted under greenhouse conditions with two different soil types, an acidic (pH = 6.3) and an alkaline (pH = 8.0) soil with different organic matter content, in a completely randomized design. The results indicated higher Se content in berseem cultivated on acidic soil. It was also observed a significant reduction (~ 45%) in plant biomass of lettuce in the acidic soil combined with Se application. The results showed that leaf Se content was negatively correlated with soil organic matter. The decreased Se content in plants cultivated on the alkaline soil with high organic matter content support that the effect of pH on Se uptake decreased as the soil organic matter content increased.

## 1. Introduction

Selenium (Se) is considered a mineral micronutrient, having characteristic physical and chemical properties, which are intermediates of metals and non-metals [1]. In the natural environment, Se occurs in four oxidation states with valences 6+, 4+, 2- and 0 (elemental form). The Se effects on human health were identified for the first time in 1957, almost a century after its discovery. It has been identified as an essential trace element for humans and animals and its adequacy may prevent hepatic necrosis due to lack of vitamin E, cardiovascular disease, muscle disorders, cancers, and also seems to play an important role in male fertility [2,3,4]. However, since the difference between Se dietary deficiency (< 40 μg/day) and toxicity (> 400 μg/day) is narrow, human and animal intake requires careful control. In addition to the beneficial effects on human health, Se is an important trace element for the daily diet of the animals, too. A lack of Se can significantly affect production efficiency and animal health, with a high mortality rate among offspring, resulting from degenerative changes in myocardium. Selenium is found in trace amounts in the Earth’s crust and mantle and characterized by unequal distribution worldwide. In soils, Se may be present naturally because of weathering processes ranging between 0.01 and 2 mg/kg, with a world mean of 0.4 mg/kg and/or anthropogenic activities, such as mining and refining processes of sulfide ores. Soil in many parts of the world has low Se concentrations (< 0.6 mg/kg), including large areas of Scandinavia, North America, New Zealand, Australia and China [5]. The lack of Se in soils and the crops grown on these soils results in approximately 500–1000 million people worldwide being affected by Se malnutrition. This makes them susceptible to health problems, such as growth retardation, immune dysfunction and increases the risk of cancer and lymphoma [6,7]. Se concentration in sedimentary rocks, especially shales and coal is much higher than in igneous rocks [8]. In Greece, the Se content in sulfide ores of Cyprus type exhibits a wide variation from a few tens to hundreds mg/kg in the Othrys, Veria and Argolis ophiolites while in the Pindos (Kondro) ophiolite complex the Se content ranges from 160 to 1900 mg/kg (average 990 mg/kg) [9,10]. The mobility and bioavailability of soil Se depends by complex chemical and biochemical process and is controlled primary by the redox potential (Eh), the percentage of soil organic matter (SOM), the clay percentage and the soil acidity (pH) [11].

The plant species constitute a link between soil types and animal species on the uptake and transfer of Se to the higher nutritional levels. Plants according to their ability to accumulate Se in their tissues are divided into three major categories: i) the hyperaccumulators plants, with concentration levels ranging from 1000 to 10,000 mg/kg, ii) the secondary accumulators or indicators, which rarely exceed a few hundred to a few thousand mg/kg Se concentration in their tissues; and iii) non-accumulators (types of grass, trees and some weeds), which generally accumulate less than 25 mg/kg [12,13].

Plants grown in soil fertilized with Se will be enriched in Se, and these plants have a critical role in transferring Se from soil into the food chain, either be used directly for human consumption, or as animal feed. The effectiveness of that common management practice can produce Se-rich products that may be regarded as functional foods. For example, Se fertilization has been proved to be an efficient method of increasing Se content in forages and cereals [14]. Since the most important source of Se is diet, in recent years, agronomic Se-biofortification strategies have been proposed as an effective and safe mean to increase human and animal Se intake. Finland has conducted a Se fertilization program, where addition of 15 mg Se/kg in multi-element fertilizers has been mandatory since 1984 [15]. On the other hand, since a limited fraction of applied Se is utilized by plants, usually less than 10%, Se overfertilization may lead to leaching and contamination of groundwater or surface water via runoff. In this context, gaining a comprehensive understanding of the behavior of added Se in soil-plant system is necessary under different pedoclimatic environments.

According to Gupta and Gupta (2017) [16], Greece are reported to have Se deficient areas, whereas Se containing fertilizers with added soluble selenate or selenite salts are, neither imported, nor manufactured in the country. Nevertheless, the mean Se content of hard and soft wheat, barley, oats, rye and corn from almost 100 different locations of Greece was 0.29 ± 0.19, 0.21 ± 0.12, 0.16 ± 0.10, 0.14 ± 0.10, 0.19 ± 0.10, and 0.12 ± 0.08 mg/kg (on a dry weight basis), respectively [17].

In this context, the aim of this study was to evaluate the effect of selenate (SeO_4_^2-^) applied as inorganic fertilizer on the biomass and Se uptake of two different crops, *Lactuca Sativa* L. (vegetable crop) and *Trifolium alexandrinum* L. (animal-feed crop) grown on two different soil types (acidic and alkaline soil) under greenhouse conditions. Moreover, this study highlights the role of soil properties, i.e., pH and SOM content in influencing Se concentration in lettuce and berseem plants for human and animal nutrition.

## 2. Results and Discussion

The properties of the two studied soils at the start of the experiment are summarized in Table 1. 

The soils are fine textured with contrasting chemical properties. Soil organic matter levels are medium to high in both soils, but over double in alkaline soil, which contains also significant amounts of carbonates as expected. Compared to alkaline soil, the concentrations of exchangeable cations are lower in acidic soil.

**Figure 1:**
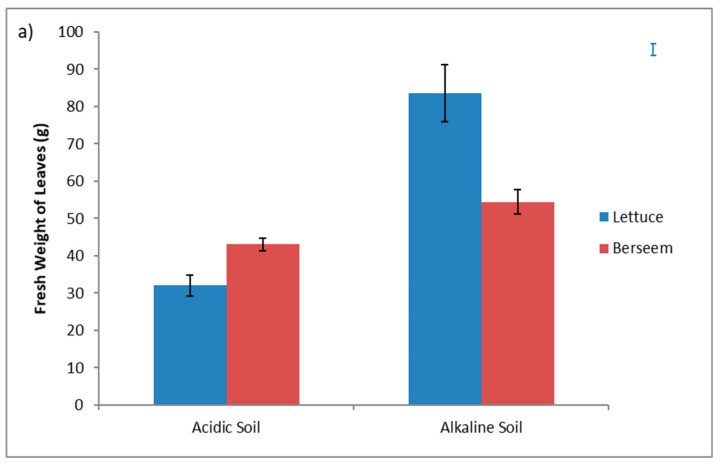

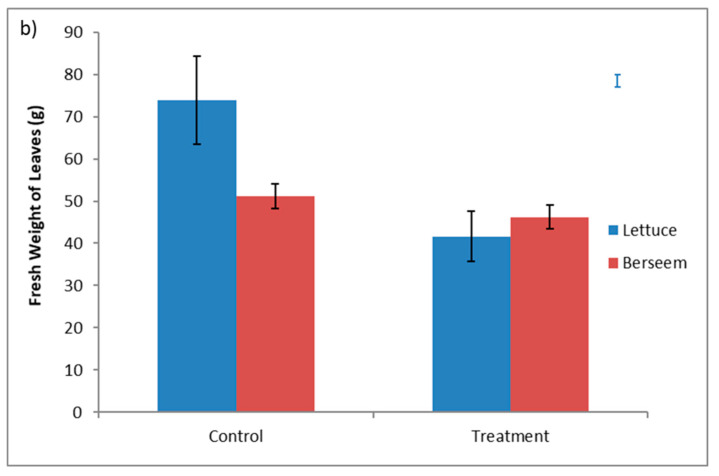
Effects of various factors on fresh weight of leaves; (**a**) cultivation vs. soil type; (**b**) cultivation vs. Se treatments. In each column the bar on it represents the standard error of the mean. Bars at the right side of the graphs are the standard error of the ANOVA analysis.

In the greenhouse experiment, lettuce growth was clearly poorer in the pots filled with acidic soil than in the pots filled with the alkaline. Aboveground biomass (fresh weight) in the acidic soil was close to 1/4 the amounts of those in the alkaline soil. This poor plant growth was most likely due to less favorable conditions in the acidic soil that developed during cultivation. According to the ANOVA results in Table 2, all the experimental factors have a marked impact on the fresh weight of leaves. Soil type and Se treatment significantly affected the fresh weight of leaves at *p* < 0.001, while cultivation effect is significant at *p* < 0.01. As shown in Figure 1a, a significant negative relationship of soil pH (as expressed by soil type) with the fresh weight of lettuce was found, suggesting that under acidic conditions the growth of lettuce was significantly restricted. Conversely, for berseem crop the effect of soil type was less important.

Lettuce plants exposed to sodium selenate exhibited a 45% decrease in fresh weight, when compared with control plants, while the reduction for berseem plants was only 10% (Figure 1b). Our results are in accordance with Ramos et al. [18] who observed an inhibitory effect of Se when lettuce plants treated with high concentrations of sodium selenate (> 16 μmol/L). Mora et al. [19], observed that the applications > 40 g Se/ha decreased the shoot growth of white clover (*Trifolium repens* L.) by 22%, whereas application at the rate of 60 g Se/ha resulting to a decrease by more than 60% compared to control plants. Several studies have also shown a significant decrease of plant growth in different crops with increase in Se plant content. For example, Soltanpour and Workman [20], reported a decrease by 10% in alfalfa yield and Banuelos et al. [21], indicated that yield of canola, barley and indian mustard decreased by 25%, 20%, and 16%, respectively, grown on Se treated soil.

**Figure 2:**
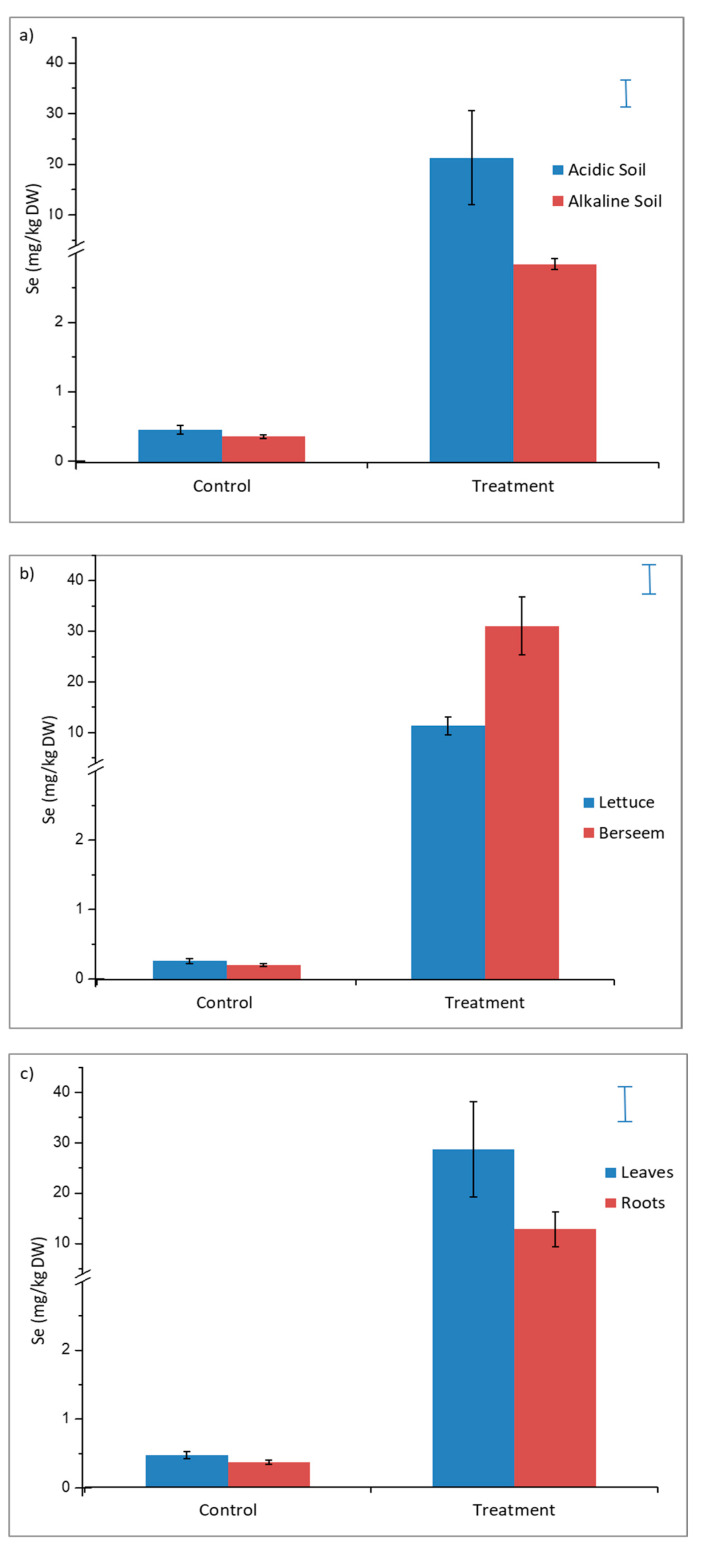
Effects of Se treatments (Control – Treatment Se); and (**a**) soil type; (**b**) cultivation; and (**c**) tissues independently of cultivation on plant Se content (mg/kg dry weight) In each column the bar on it represents the standard error of the mean. Bars at the right side of the graphs are the standard error of the ANOVA analysis.

The results of the performed ANOVA showed the effects of Se application, soil type, cultivation, and tissues on Se plant content. (Table 3). In treated samples, the Se plant content independently of the cultivation, was higher in the acidic than the alkaline soil, whereas the control plants were not affected by the soil type (Figure 2a). Dimes et al. [22], reported that maximum Se uptake by *Trifolium subterraneum* was observed at pH 6.7.

Se fertilization has been shown to significantly enhance Se concentrations in lettuce and berseem plant and variance analyses indicated that the increased Se concentration of the treated plants was statistically significant, when compared to the control samples (Figure 2b).

The form of Se added, selenate, is weakly adsorbed by electrostatic forces of attraction forming outer-sphere surface complexes, and therefore, is more exchangeable, resulting in higher plant availability and potential for leaching in relation to selenite. Se concentrations in lettuce leaves ranged between 2 and 17 mg/kg dry weight, whereas for roots the treatment resulted in Se concentrations between 1.8 and 7.5 mg/kg dry weight. Se concentrations in berseem leaves ranged between 4 and 54 mg/kg dry weight while for roots the Se addition resulted in Se concentrations between 3.0 and 18 mg/kg dry weight. These results indicated that the Se accumulation in plant tissues was higher in berseem than lettuce plants (Figure 2b), due to the ability of Fabaceae to absorb high levels of Se [23]. Fabaceae species have an elevated protein content in relation to Compositae *(Lactuca Sativa* L.) and Se in these species can substitute for S in protein amino acids (cysteine and cystine), and therefore increased Se contents [24].

Apart from plant genotype, another factor that actively participates in the uptake of Se is the shape and type of the root system of plants. *Lactuca sativa* is characterized by tap root system, while *Trifolium alexandrinum* plants have fibrous root system, which occupies almost the whole soil volume, resulting in the increased possibility of Se uptake.

The high plant Se concentrations of this study are comparable with Se concentrations of plants grown on naturally Se-rich (seleniferous) soils. For example, Dhillon et al. [25], reported Se concentrations (mg Se/kg dry weight) of 1.5–86.6 for raya (*Brassica juncea* Czern L.), 0.7–58.3 for wheat (*Triticum aestivum* L.) 0.7–58.3 for maize (*Zea mays* L.) and 1.5–4.6 for rice (*Oryza sativa* L.) when grown in alkaline seleniferous soils in north-western India. Recently, Fan et al. [26] observed that the addition of 30 mg/kg selenate increased the Se contents of leaves and roots of tobacco to 312.61 and 242.28 mg/kg, respectively. For plant tissues, controls showed the same amount of Se independently cultivation. The above observations lead to the conclusion that significant changes in the Se concentrations of plant tissues, occurs in soils enriched with Se. Application of selenate to the soil resulted in a significant increase in the Se concentration in leaves, whereas the Se increments in root tissues were lower (Figure 2c). Several studies demonstrated that Se is mainly accumulated in plant shoot than in root [27]. As previously suggested by Krystofova et al. [28], a high Se content in leaves shows that inorganic Se is intensively transported from roots by the xylem to photosynthetically active organs. Moreover, according to Zhang [29] selenate forms like those used in our study are more easily transported to shoots, while selenite tends to accumulate in plant roots.

The influence of soil properties on leaf Se concentrations was investigated. Table 4 shows the negative relationship between SOM and plant Se content for lettuce and berseem cultivation. The correlation was strong in lettuce (r = −0.86), but a lower content of SOM was strongly negatively correlated to a higher content of Se in the berseem (r = −0.97).

Soil organic matter, as an important soil component that retains Se, plays a very significant role in Se immobilization and influence the availability of Se to plants, especially in acidic soils [30,31]. The interaction of Se and soil organic matter may occur via various mechanisms: (i) Complexation of Se by high affinity sorption sites of organic matter [32]; (ii) microbial reduction to lower oxidation state enhancing incorporation into low-molecular-weight humic substances, proteins and amino acids [31]; and (iii) indirect complexation of Se by organic matter-metal complexes [32,33,34].

**Figure 3:**
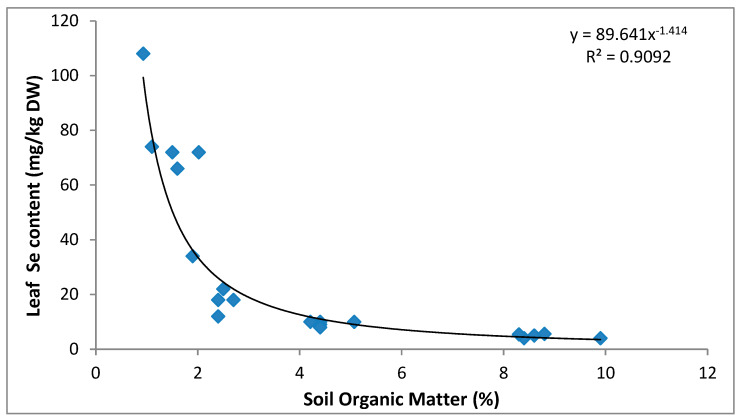
Relationship between soil organic matter and Se content of lettuce and berseem crops grown in soils with a pH range of 6.1 to 8.1 amended with sodium selenate.

Sorting the data for the acidic and alkaline soils with respect to SOM levels allowed a comparison of the effect of SOM on Se uptake. Our results indicated that up to 90% of the variation in plant Se content in the greenhouse experiment could be explained by SOM content (Figure 3.). For both soil types, plants grown in the soils with the lower organic matter content took up more Se than those grown in the soils with high organic matter content. According to Table 5, an increase in SOM content from 1.4 to 4.5% led to a decrease in Se uptake from 78.4 to 9.4 mg/kg dry weight, representing a total decrease up to 88% for berseem cultivation. Similarly, Johnsson [11] reports that at soil pH 7, an increase of SOM from 1.5 to 39% resulted in significant decrease in Se uptake of 88%, and 69% for wheat grain and rape, respectively.

More recently, Ferri et al. [35] found in a sandy alkaline soil amended with selenate, a significant reduction in Se content of lettuce leaves after the addition of organic substances (polysaccharide – carboxymethylcellulose). Moreover, several studies have shown that Se applied into the soil are quickly immobilized by SOM forming water-insoluble complexes with organic compounds, and thus, less plant available Se ([31] and references therein). However, as previously suggested by Natasha et al. [27], very limited data is available regarding the speciation and molecular structure of Se bound to organic matter.

Ammonium bicarbonate-diethylenetriaminepentaacetic acid (AB-DTPA) extractable Se in the soil was related to pH, with very low content for the acidic soil (< 0.004 mg/kg), while in alkaline soil high available Se values were observed. According to Dhillon et al. [25], the ability of a certain extractant in evaluating the soil Se availability to plants depends on the type and nature of the soil.

**Figure 4:**
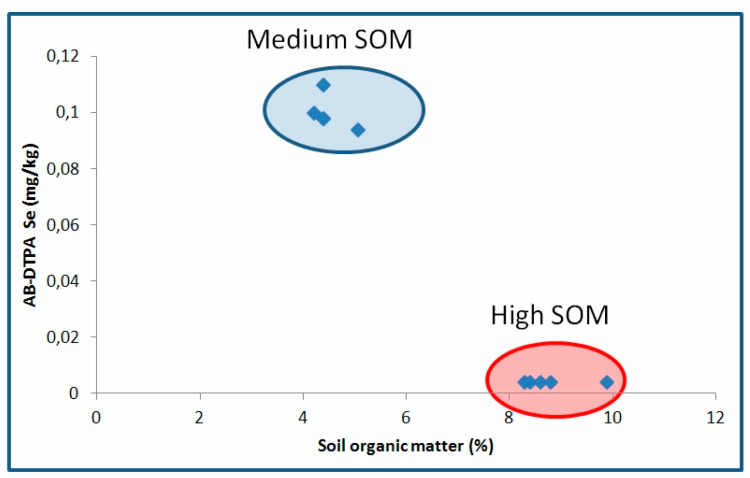
Relationship between available Se (AB-DTPA) and soil organic matter (SOM) content in the alkaline soil amended with sodium selenate.

Soltanpour and Workman [20] found that AB-DTPA can be used as a reliable of Se availability and toxicity reporting at the same time that AB-DTPA extracted about 1.5 times more available Se than hot water. According to their findings AB-DTPA Se > 0.1 mg/kg increased the Se content in alfalfa plants above of 5 mg/kg. Ylaranta [36] reported extractable with hot water, soil Se concentrations of 2 – 27 μg/kg, concentrations considered to be too low to produce crops with sufficient Se for human nutrition [37]. In our study, although no differences in available Se concentration between the crops in the acidic soil were observed, the high pH values of the alkaline soil with medium organic matter content, led to an increase in Se availability (Figure 4).

In this case, the available Se concentration ranged from 0.094 – 0.11 mg/kg which was close to the WDEQ-LQD [38] marginal available Se level. At the highest organic matter level, however, available Se was similar to the acidic soil (< 0.004 mg kg) as Se bioavailability rapidly decreases with increasing SOM content. Dhillon et al. [39] after greenhouse and field experiments concluded that the application of organic manures is as effective way to decrease the bioavailability of Se reducing the transfer of Se from soil to plant in seleniferous soils.

## 3. Materials and Methods

The experimental procedure was carried out in a greenhouse of the Agricultural University of Athens, under controlled environmental conditions. The test plants were *Lactuca sativa* L. and *Trifolium alexandrinum* L., as consumption’s indicators of human, and animals, respectively. Vegetables were found to provide more than 85% of the average daily human dietary Se intake in seleniferous soils. Since lettuce is the most consumed leafy plant in many parts of the world, this crop can be used in as an efficient way of increasing intake of Se by humans. Two soil types (an alkaline amended with peat and acidic one) with different content of soil organic matter (Table 1) were chosen and polyethylene pots were filled with 2 kg of each of one with daily irrigation to keep soil moisture at 70% field capacity. The chemical form of Se was the sodium selenate (Na_2_SeO_4_ 1 M) at the high dose of 4 mg Se/kg as previously indicated by Hawkesford and Zhao [40]. As a control, plants grown without Se application were used. Moreover, no NPK fertilization was given during the experiment. The experiment was a completely randomized block design with three replicates for control plants and five replicates for treatment plants, totaling 32 pots.

In order to assess the effect of soil properties on Se uptake, the soil samples at the end of experiment, were recovered by gentle shaking and scraping the roots, air dried and sieved (2 mm) to remove stones and plant residues. Particle-size distribution, calcium carbonate, pH, organic matter, and exchangeable cations were determined according standard [41]. Briefly, the soil parameters were determined as follows: pH in a soil-distilled water paste (1:1) using a glass electrode meter, particle size analysis according to the Bouyoucos method, organic matter using the wet oxidation method with K_2_Cr_2_O_7_ – H_2_SO_4_ and FeSO_4_.7H_2_O titration method and the exchangeable cations, according to the method of CH_3_COONH_4_. Moreover, since P influence the Se uptake [42], the available P was determined according to the Olsen method. Ammonium bicarbonate-diethylenetriaminepentaacetic acid (AB-DTPA) extractable Se was determined according to Soltanpour and Workman [20] method. Briefly, 10 g soil was shaken with 20 mL of 1 M NH_4_HCO_3_ and 0.005 M DTPA for 15 min on a reciprocating shaker, the soil suspension was centrifuged, and the supernatant was analyzed for Se. In most cases, the values of soil properties actually found after the experimental period, were different from the initial ones as a result of the effect of cultivation. This differentiation was more pronounced for soil organic matter values in the alkaline soil amended with peat under lettuce cultivation. Similarly, Kukkonen et al. [43] found statistically significant effects of crop plant (*Carum carvi* L.) on properties in a silt clay soil amended with peat.

At harvest the plants were separated into shoots and roots, followed washing with deionized water and placing in a laboratory oven for three days at approximately 60 ^o^C and then ground to a fine powder. For determination of Se, due to its high evaporating ability, the autoclave combustion of high pressure was used. The sample (0.5 g) was placed in cylindrical porcelain capsule, with 1 mL of hydrogen peroxide (H_2_O_2_) and 6 mL of concentrated nitric acid (HNO_3_). The solutions were transferred to volumetric flasks (50 mL) and the Se was determined by the ICP-MS spectrometry according to ASTM D5673 – 10 standard method [44].

Data processing performed by ANOVA at 95% confidence using StatGraphics Centurion for Windows software as described by Petridis et al. [45].

## 4. Conclusions

Selenium fertilization of lettuce and berseem (*Lactuca Sativa* L. and *Trifolium alexandrinum* L., respectively) grown on an acidic and alkaline soil increased the plant Se content and, potentially, dietary intake of Se by humans and animals. Our results indicated that the effect of pH on Se uptake decreased as the soil organic matter content increased. In alkaline soils with medium levels of soil organic matter, a larger part of the Se, in the form of added selenate, seems to exist in a plant-available form, because an increase in pH decreases the amount of pH-dependent binding sites, i.e., the positive charges on clay minerals and sesquioxides. However, an increase in SOM from 4.5% to 8.5% in the alkaline soil, led to a decrease in Se availability indicating that high levels of organic matter can restrict the role of soil pH of Se absorption by plants. Therefore, in accordance with earlier findings, raising the soil pH does not seem to be a suitable management practice increasing Se availability of soils rich in organic matter. The original Se concentration of both soils had no effect on Se uptake by lettuce and berseem whereas the application of sodium selenate results in higher Se content in berseem in relation to lettuce. Since additional soil properties such as redox potential, competing anions and microbial activity greatly affect Se availability, further studies are needed to confirm the effectiveness of Se fertilization strategy under field conditions and to elucidate the main mechanisms of Se uptake by plants.

## Figures and Tables

**Table 1:** Selected physicochemical properties of the soils at the start of the experiment.

Parameter	Acidic Soil	Alkaline Soil
Texture	Clay	Clayloam
CaCO_3_ (%)	-	20.4
Organic matter (%)	2.1	5.0
pH (1:1 s/w)	6.3	8.0
Exchangeable K (cmol(+)kg^−1^)	0.33	4.58
Exchangeable Ca (cmol(+)kg^−1^)	1.73	5.35
Exchangeable Mg (cmol(+)kg^−1^)	0.16	3.70
Available P (mg/kg)	12	65
Available Se (mg/kg)	< 0.004	< 0.004

**Table 2:** Analysis of Variance for effects of various factors on fresh weight of leaves.

Source	Sum of Squares	Df	Mean Square	F-Ratio	*p*-Value
MAIN EFFECTS^1^					
A:Cultivation	617.44	1	617.44	10.32	0.0036
B:Soil	7416.7	1	7416.7	123.97	0.0000
C:Treatment	2597.77	1	2597.77	43.42	0.0000
INTERACTIONS					
AB	3231.68	1	3231.68	54.02	0.0000
AC	1410.42	1	1410.42	23.57	0.0001
BC	890.53	1	890.53	14.88	0.0007
RESIDUAL	1495.69	25	59.8275		
TOTAL (corrected)	16480.9	31			

^1^ Cultivation (Lettuce – Berseem). Soils (Acidic – Alkaline). Treatments (Control – Treatment Se).

**Table 3:** Analysis of Variance for effects of various factors on Se content.

Source	Sum of Squares	Df	Mean Square	F-Ratio	*p*-Value
					
MAIN EFFECTS^1^					
A:Cultivation	1587.75	1	1587.75	17.06	0.0001
B:Soils	2733.08	1	2733.08	29.37	0.0000
C:Tissues	871.347	1	871.347	9.36	0.0035
D:Treatments	4767.74	1	4767.74	51.23	0.0000
INTERACTIONS					
AB	1602.0	1	1602.0	17.22	0.0001
AC	603.931	1	603.931	6.49	0.0138
AD	1676.4	1	1676.4	18.01	0.0001
BC	1594.01	1	1594.01	17.13	0.0001
BD	2726.33	1	2726.33	29.30	0.0000
CD	807.767	1	807.767	8.68	0.0048
RESIDUAL	4932.04	53	93.0574		
TOTAL (Corrected)	27369.3	63			

^1^ Cultivation (Lettuce – Berseem). Soils (Acidic – Alkaline). Tissues (Leaves – Roots). Treatments (Control – Treatment Se).

**Table 4:** Correlation between leaf Se content and soil organic matter (SOM) content for lettuce and berseem plants.

	SOM	Se Content
**Lettuce**		
SOM	1	−0.86**
Se content	−0.86**	1
**Berseem**		
SOM	1	−0.97***
Se content	−0.97***	1

** *p* < 0.01, *** *p* < 0.001.

**Table 5:** Soil organic matter and Se content (mg/kg dry weight) of lettuce and berseem crops grown in soils with a pH range of 6.5 to 8.1 at the end of the experiment. The mean ± SE of five replicates is given.

Soil Type	Cultivation	Soil Organic Matter(%)	Se content(mg/kg DW)
Acidic	Lettuce	2.4 ± 0.1	20.8 ± 3.7
	Berseem	1.4 ± 0.9	78.4 ± 7.5
Alkaline	Lettuce	8.5 ± 0.3	4.8 ± 0.3
	Berseem	4.5 ± 0.2	9.4 ± 0.4
